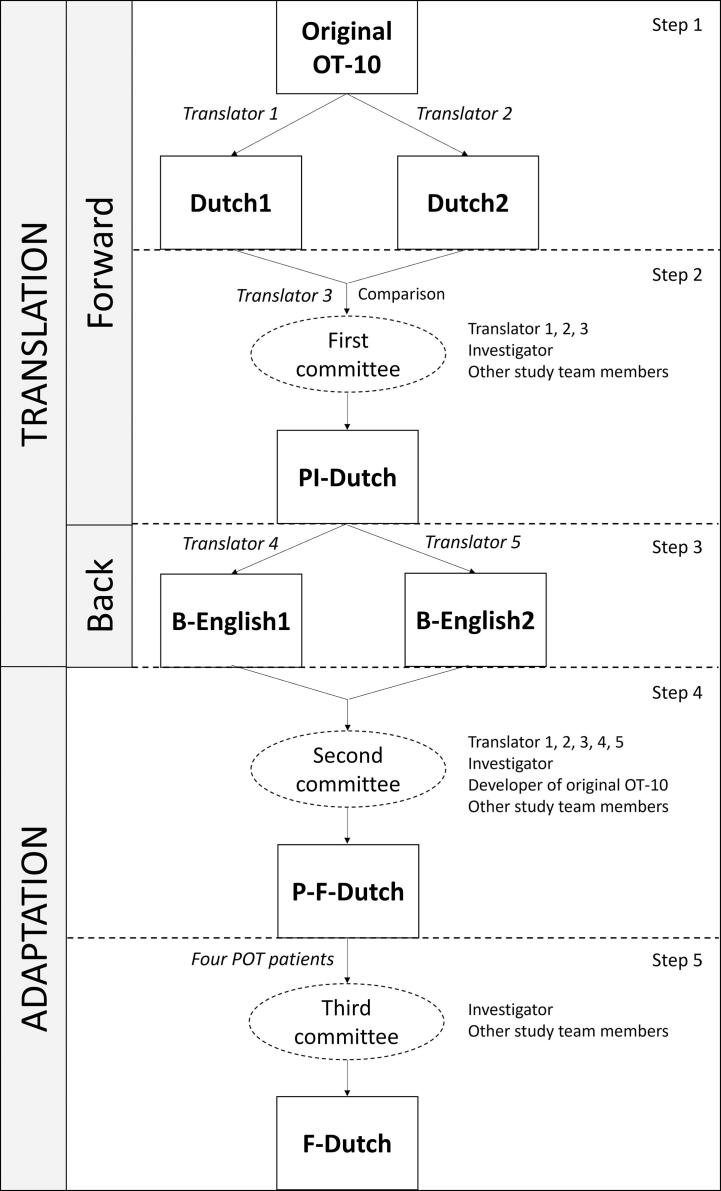# Dutch translation, adaptation and validation of the OT-10 scale for orthostatic tremor

**DOI:** 10.1016/j.prdoa.2023.100200

**Published:** 2023-05-18

**Authors:** B.E.K.S. Swinnen, M. Bergers, W.A. Babeliowsky, D. Torres-Russotto, R.M.A. de Bie, A.F. van Rootselaar

**Affiliations:** a Department of Neurology and Clinical Neurophysiology, Amsterdam University Medical Centers, Amsterdam Neuroscience, University of Amsterdam, Amsterdam, The Netherlands; b Department of Neurological Sciences, University of Nebraska Medical Center, Omaha, Nebraska, USA

**Keywords:** Orthostatic tremor, OT-10 scale, Dutch translation, Adaptation, Validation

## Abstract

**Introduction:**

There are currently no effective treatments for primary orthostatic tremor (POT). An adequate disease-specific POT severity scale is a prerequisite to conduct clinical trials and monitor disease severity in clinical practice. Recently, the English OT-10 scale has been developed for this purpose. Here we aimed to obtain a scale to measure the severity of POT in Dutch speaking individuals.

**Methods:**

An established translation, adaptation and validation approach was employed to obtain a Dutch version of the OT-10 scale. Validation was performed in a Dutch POT cohort (n = 46).

**Results:**

A Dutch OT-10 scale was obtained which showed good internal consistency (Cronbach’s alpha > 0.80), total score test–retest reliability (intraclass correlation coefficient > 0.80), and concurrent validity (Pearson correlation > 0.80). Item-to-total correlation was good (weighted kappa > 0.40) for all items, and item test–retest reliability was good (weighted kappa > 0.40) for eight out of ten items. Overall, the Dutch OT-10 scale demonstrated acceptable validity.

**Conclusions:**

We obtained and validated a Dutch version of the OT-10 scale, capturing POT severity. Next to its use in clinical practice, translation and validation of the OT-10 scale in more languages will help to find evidence-based treatments for POT.

## Highlights

• An adequate OT severity scale is needed for clinical trials and clinical practice.
• Through translation and adaptation, we obtained a Dutch version of the OT-10 scale.
• The Dutch OT-10 scale demonstrated acceptable validity in a Dutch OT population.

## 1 Introduction

Primary orthostatic tremor (POT) is a rare neurological disorder characterized by tremor in the legs (and other body parts) upon standing leading to difficulties in maintaining balance [1], [2], [3]. Because standing still is essential in many activities of daily living (e.g., cooking, showering, and waiting in line) POT leads to severe impairments. Currently there is no cure and treatment consists of (largely ineffective) symptomatic treatments including medication and physiotherapy [4]. Clinical trials evaluating possible symptomatic treatments demand an adequate severity scale that adequately captures the difficulties patients experience in their daily life. For POT however, generic (e.g. clinical global impression) and/or physician-based (like MDS-UPDRS III in Parkinson’s disease) severity scales are inadequate since severity in POT can only be measured based on subjective disease-specific symptoms. Hence, a disease-specific patient-reported scale in the patient’s mother tongue is pivotal. Recently, the OT-10 scale, which consists of ten questions, has been developed for this purpose and validated in an English speaking population [5]. In order to obtain an adequate scale to measure the severity of POT in Dutch speaking individuals we aimed to translate, adapt and validate the OT-10 scale.

## 2 Methods

An established translation, adaptation and validation approach was employed to obtain a Dutch version of the OT-10 scale [6].

### 2.1 Translation

The English OT-10 scale (freely accessible as ‘Supplementary material 3’ in the original publication [5]) was translated into Dutch (*i.e.*, forward translation) by two independent native Dutch speaking bilingual translators (Supplementary Fig. 1). The first translator was acquainted with health care terminology and was familiar with orthostatic tremor. The second translator was not familiar with health care terminology nor orthostatic tremor, but was acquainted with Dutch informal phrases, health care slang, idiomatic expressions and emotional terms. At the end of this step two initial forward-translated versions were obtained; ‘Dutch1’ and ‘Dutch2’.

In a second step Dutch1 and Dutch2 were compared with the original English version of the OT-10 scale by a third bilingual independent translator without specific qualifications (Supplementary Fig. 1). All ambiguities and discrepancies were discussed and resolved using a committee approach. This first committee included the first, second and third translator, the principal investigator (B.S.) and other members of the research team. At the end of this step a preliminary initial Dutch version of the OT-10 scale was obtained; ‘PI-Dutch’.

As a third step PI-Dutch was translated back into English by two independent fluently English speaking bilingual translators; the fourth and the fifth translator (Supplementary Fig. 1). Both translators were completely blind to the original version of the OT-10 scale. The fourth translator had similar qualifications and characteristics as the first translator; *i.e.*, acquainted with health care terminology and familiar with orthostatic tremor. The fifth translator had similar qualifications and characteristics as the second translator; *i.e.*, not familiar with health care terminology nor orthostatic tremor, but acquainted with English informal phrases, health care slang, idiomatic expressions and emotional terms. At the end of this step two back-translated versions of the OT-10 scale were obtained; ‘B-English1’ and ‘B-English2’.

### 2.2 Adaptation

In a fourth step these back-translated English versions were compared with each other and with the original English version by a second committee including all five translators, the principal investigator, other study team members as well as one of the developers of the original OT-10 scale (D.T.-R.) (Supplementary Fig. 1). Ambiguities and discrepancies regarding cultural meaning, informal phrases and idioms in words and sentences were discussed and resolved. During this discussion the principal investigator made adaptations to the ‘PI-Dutch’ version through consensus. At the end of this step a pre-final Dutch version of the OT-10 scale was obtained; ‘P-F-Dutch’.

In step five of the process, this ‘P-F-Dutch’ underwent pilot testing in a set of four Dutch speaking POT patients, to obtain input about the clarity and relevance of the scale items (Supplementary Fig. 1). These patients were asked to rate the instructions and items of the ‘P-F-Dutch’ using a dichotomous scale (‘clear’ or ‘unclear’). If ‘unclear’ was indicated, the participant was asked to provide suggestions how to make the language clearer. These suggestions were discussed in a third committee meeting involving the principal investigator and other study team members. During this committee meeting the principal investigator adapted the ‘P-F-Dutch’ through consensus. At the end of this step the final Dutch version of the OT-10 scale was obtained; ‘F-Dutch’.

### 2.3 Validation

As a sixth step, a cohort of POT patients were asked to fill in the final Dutch version of the OT-10 scale twice. All patients fulfilled POT diagnostic criteria, with sufficient exclusion of alternative causes [1], [2]. These data were entered in the Amsterdam OT database, which is a monocentric database assembling medical information of all POT patients evaluated at Amsterdam UMC location AMC. For the purpose of this research, information regarding demographics, OT features, and OT treatment was extracted from this database. Based on multi-domain questionnaires and medical information in this database, for each patient clinical global impression (CGI) was estimated by one author (B.S.). The generation and use of this database is according to local regulations and has received a waiver for active consent by the local ethical committee. Nevertheless, patients were informed that OT-10 administration was performed for the purpose of validation.

In a final seventh step, the obtained data concerning Dutch OT-10 scale test–retest and CGI were analysed to assess the validity of the scale in the Dutch POT population. These analyses were largely in line with those performed to develop the original OT-10 scale, but were slimmed down to the following essential analyses: internal consistency, item-to-total correlation, total score test–retest reliability, individual item test–retest reliability, and concurrent validity [5]. As a measure of internal consistency, Cronbach’s alpha was employed with a minimal recommended cut-off of 0.80. For item-to-total correlation a minimal recommended cut-off of 0.40 was used. Total score test–retest reliability was calculated using a two-way random intraclass correlation coefficient (ICC), for both single and average measures. For individual item test–retest reliability we used weighted kappa statistic with linear weighting and a minimal recommended cut-off of 0.40. Concurrent validity was applied to test for an agreement between the Dutch OT-10 and a similar measure, the CGI. The latter is a generic 7-point severity scale, broadly used in clinical research. Regarding sample size determination, a 4.5:1 subject-to-item ratio was employed [7]. All analyses were conducted in R (R Core Team (2022). R: A language and environment for statistical computing. R Foundation for Statistical Computing, Vienna, Austria, https://www.R-project.org/).

## 3 Results

### 3.1 Translation

The translation and adaptation phases were conducted successfully and without notable issues. The most notable ambiguities to resolve in step two of the translation process encompassed; establishing the most adequate translation of the phrases ‘unsteadiness’, ‘shaking’, and ‘need to lean against something or someone’, as well as establishing a common most optimal backbone of all ten question sentences. At the end of step five the final Dutch version of the OT-10 scale was obtained (Supplementary File 1).

### 3.2 Validation

The POT validation cohort’s characteristics regarding demographics, POT features, POT treatment and CGI are displayed in Table 1. The Dutch OT-10 was administered in 46 POT patients, data of which were used to assess internal consistency, item-to-total correlation and concurrent validity. In 42 of them, the Dutch OT-10 was administered a second time with a mean interval of 25.3 days (SD 9.8), data of which were used to assess test–retest reliability. Internal consistency of the Dutch OT-10, as measured by Cronbach’s alpha, was good (alpha = 0.83 – Table 2). Item-to-total correlation was acceptable (*i.e.*, >0.40) for all individual items (Table 2). Total score test–retest reliability was good (ICC = 0.89) and very good (ICC = 0.94) for both single as well as average measures respectively (Table 2). Individual item test–retest reliability was good (>0.40) for all items, except for item four and nine (Table 2). Concurrent validity, measured by the correlation between the Dutch OT-10 and CGI, was good (0.778 – Table 2).

**Table 1:** Patient characteristics of the Dutch OT-10 validation cohort. CGI, clinical global impression; EMG, electromyography; POT, primary orthostatic tremor; SD, standard deviation; y, years; *, can include combination of medications.

Patients – n	46	
Male/female	17/29	
Mean age – y (SD)	69.7 (8.9)	
Mean age at POT onset – y (SD)	54.7 (10.9)	
Mean POT disease duration – y (SD)	15.0 (8.4)	
POT EMG confirmation – n	40	

Current medication for POT* – *n*		
	Clonazepam	19
	Perampanel	17
	Gabapentin	2
	Propranolol	1
	None	17

*CGI* – *n*		
	1	0
	2	2
	3	7
	4	15
	5	18
	6	3
	7	1

*OT-10*		
	Test-retest – n	42
	Test-retest interval – days (SD)	25.3 (9.8)

**Table 2:** Statistical analyses regarding validation of Dutch OT-10. ^1^ Cronbach’s alpha, ^2^ two-way random intraclass correlation coefficient, ^3^ weighted Kappa, ^4^ correlation between Dutch OT-10 and CGI (clinical global impression), ^5^ single measures, ^6^ average measures, *p < 0.0005.

	Internal consistency^1^	Item-to-total correlation	Total test–retest reliability^2^	Individual item test–retest reliability^3^	Concurrent validity^4^
Total scale	0.829		0.89*^5^ 0.94*^6^		0.778*
Items					
1		0.678		0.703	
2		0.807		0.561	
3		0.879		0.558	
4		0.415		0.180	
5		0.614		0.628	
6		0.521		0.760	
7		0.549		0.726	
8		0.640		0.408	
9		0.570		0.351	
10		0.613		0.593	

## 4 Discussion

Following a rigorous translation and adaptation approach, we were able to obtain a Dutch version of the OT-10 scale. Upon testing in a Dutch POT cohort of comparable magnitude (n = 46) as for the English OT-10 (n = 54), the Dutch OT-10 showed good overall validity [5]. Moreover, values regarding internal consistency, item-to-total correlation, total test–retest reliability, and concurrent validity are similar to the ones for the English OT-10 [5]. Only regarding individual item test–retest reliability, scores are generally lower (around 0.50) than for the English OT-10 (around 0.70), with two items scoring below the recommended cut-off [5]. This is most likely related to a longer test–retest interval (25 days versus 14 days) for the Dutch OT-10 [5]. Given the very good overall test–retest reliability (as well as other validity measures), we believe that the validity of the Dutch OT-10 is uncompromised.

Limitations of this study include the lack of a large number of patients in the validation cohort because of the rarity of the disease, as well as the notion that despite all measures taken a perfect translation is hard to obtain due to the differences in culture and language uses.

Altogether, we were able to obtain a validated POT severity scale to be used in Dutch speaking individuals. This Dutch OT-10 scale can be an important outcome measure in therapeutic clinical trials and other POT-related research. Moreover, it’s a useful tool in clinical practice to monitor treatment effect in an individual POT patient. Translation and validation of the OT-10 scale in many more languages will be pivotal to propel POT research globally.

## CRediT authorship contribution statement

**B.E.K.S. Swinnen:** Conceptualization, Methodology, Formal analysis, Investigation, Writing – original draft. **M. Bergers:** Methodology, Formal analysis, Investigation, Writing – review & editing. **W.A. Babeliowsky:** Investigation, Writing – review & editing. **D. Torres-Russotto:** Investigation, Writing – review & editing. **R.M.A. de Bie:** Investigation, Writing – review & editing. **A.F. van Rootselaar:** Conceptualization, Investigation, Resources, Writing – review & editing, Supervision.

## Declaration of Competing Interest

The authors declare that they have no known competing financial interests or personal relationships that could have appeared to influence the work reported in this paper.

## Figures and Tables

**Supplementary figure 1:**