# Supplementary material for: Dutch translation, adaptation and validation of the OT-10 scale for orthostatic tremor
Source: Clin Park Relat Disord. 2023 May 18;9:100200. doi: 10.1016/j.prdoa.2023.100200 (PMC10213302; doi:10.1016/j.prdoa.2023.100200)
Supplement: Supplementary data 1 [file mmc1.pdf]

Orthostatische tremor schaal (OT-10)

Deze vragenlijst gaat over een gevoel van instabiliteit, ongemak, of trillen tijdens staan. Deze verschijnselen kunnen van invloed zijn op wat u kunt doen en ze kunnen de éne dag erger zijn dan de andere. Wij willen graag van u weten hoeveel last u heeft van deze klachten **gedurende een gemiddelde dag in de afgelopen week, inclusief vandaag**. Er zijn ook vragen over problemen die u misschien niet heeft en die u ook nooit zult krijgen. Kijk goed of u alle vragen beantwoord heeft; laat alstublieft geen vragen open. Lees alle antwoordopties goed door, voordat u antwoord geeft.

Selecteer alstublieft één optie. Het gaat steeds om uw gebruikelijke symptomen of algemene beleving van uw klachten over de afgelopen week, inclusief vandaag.

VRAAG 1

Gemiddeld gedurende de afgelopen week, **hoe lang nadat u bent gaan staan, ervoer u een gevoel van instabiliteit, ongemak, of trillen**?

Ik heb geen klachten tijdens staan	**0**
Na langer dan 5 minuten	**1**
Na 3 tot 5 minuten	**2**
Na 1 tot 2 minuten	**3**
Binnen 1 minuut	**4**
Meteen of bijna meteen	**5**

VRAAG 2

Gemiddeld gedurende de afgelopen week, **hoe lang kon u staan zonder steun (bijvoorbeeld zonder tegen iets of iemand aan te leunen)?**

Ik heb geen klachten tijdens staan	**0**
Langer dan 5 minuten	**1**
3 tot 5 minuten	**2**
1 tot 2 minuten	**3**
Minder dan 1 minuut	**4**
Ik kan niet staan zonder steun	**5**

VRAAG 3

Gemiddeld gedurende de afgelopen week, **hoe lang kon u praten of een gesprek voeren terwijl u stond zonder te steunen (bijvoorbeeld zonder tegen iets of iemand aan te leunen)?**

Ik heb geen steun nodig	**0**
Langer dan 5 minuten	**1**
3 tot 5 minuten	**2**
1 tot 2 minuten	**3**
Minder dan 1 minuut	**4**
Ik kan niet staan zonder steun	**5**

VRAAG 4

<u>Gemiddeld gedurende de afgelopen week, **hoe vaak was het moeilijk om met iemand te praten terwijl u stond?**</u>

Nooit	**0**
Eén keer per week	**1**
De meeste dagen	**2**
Eén keer per dag	**3**
Vaker dan één keer per dag	**4**
Altijd	**5**

VRAAG 5

<u>Gemiddeld gedurende de afgelopen week, **hoe vaak had u het idee dat u zou gaan vallen als u geen steun had?**</u>

Nooit	**0**
Eén keer per week	**1**
De meeste dagen	**2**
Eén keer per dag	**3**
Vaker dan één keer per dag	**4**
Altijd	**5**

VRAAG 6

<u>Gemiddeld gedurende de afgelopen week, **hoe vaak gebruikte u hulp om u voort te bewegen (bijvoorbeeld een wandelstok, looprek, rollator, rolstoel, of een ander persoon)?**</u>

Nooit	**0**
Eén keer per week	**1**
De meeste dagen	**2**
Eén keer per dag	**3**
Vaker dan één keer per dag	**4**
Altijd	**5**

VRAAG 7

<u>Gemiddeld gedurende de afgelopen week, **hoe vaak belette uw gevoel van instabiliteit, ongemak, of trillen u om naar buiten te gaan?**</u>

Nooit	**0**
Eén keer per week	**1**
De meeste dagen	**2**
Eén keer per dag	**3**
Vaker dan één keer per dag	**4**
Altijd	**5**

VRAAG 8

Gemiddeld gedurende de afgelopen week, **hoe vaak beperkte uw gevoel van instabiliteit, ongemak, of trillen u bij werk of bezigheden thuis?**

Nooit	**0**
Eén keer per week	**1**
De meeste dagen	**2**
Eén keer per dag	**3**
Vaker dan één keer per dag	**4**
Altijd	**5**

VRAAG 9

Gemiddeld gedurende de afgelopen week, **hoe vaak had u steun nodig (bijvoorbeeld tegen iets aanleunen, een wandgreep vasthouden, of gaan zitten) terwijl u een douche of bad nam?**

Nooit	**0**
Zelden	**1**
Soms	**2**
Meestal	**3**
Altijd	**4**
Ik kan niet zelfstandig douchen of een bad nemen	**5**

VRAAG 10

Gemiddeld gedurende de afgelopen week, **hoe vaak had u steun nodig tijdens aan- of uitkleden (bijvoorbeeld tegen iets of iemand aanleunen)?**

Nooit	**0**
Eén keer per week	**1**
De meeste dagen	**2**
Eén keer per dag	**3**
Vaker dan één keer per dag	**4**
Altijd	**5**

Hartelijk dank voor het invullen van deze vragenlijst. Vergeet niet dat een deel van de vragen betrekking kan hebben op klachten die u niet heeft en ook nooit zult krijgen.
